# A large proportion of patients with small ruptured abdominal aortic aneurysms are women and have chronic obstructive pulmonary disease

**DOI:** 10.1371/journal.pone.0216558

**Published:** 2019-05-28

**Authors:** Antti Siika, Moritz Lindquist Liljeqvist, Sayid Zommorodi, Olga Nilsson, Patricia Andersson, T. Christian Gasser, Joy Roy, Rebecka Hultgren

**Affiliations:** 1 Department of Molecular Medicine and Surgery, Karolinska Institutet, Stockholm, Sweden; 2 Department of Reconstructive Plastic Surgery, Karolinska University Hospital, Stockholm, Sweden; 3 Department of Vascular Surgery, Karolinska University Hospital, Stockholm, Sweden; 4 Department of Solid Mechanics, KTH Royal Institute of Technology, Stockholm, Sweden; NIHR Leicester Biomedical Research Centre, UNITED KINGDOM

## Abstract

**Objective:**

In a population-based cohort of ruptured abdominal aortic aneurysms (rAAAs), our aim was to investigate clinical, morphological and biomechanical features in patients with small rAAAs.

**Methods:**

All patients admitted to an emergency department in Stockholm and Gotland, a region with a population of 2.1 million, between 2009–2013 with a CT-verified rupture (n = 192) were included, and morphological measurements were performed. Patients with small rAAAs, maximal diameter (Dmax) ≤ 60 mm were selected (n = 27), and matched 2:1 by Dmax, sex and age to intact AAA (iAAAs). For these patients, morphology including volume and finite element analysis-derived biomechanics were assessed.

**Results:**

The mean Dmax for all rAAAs was 80.8 mm (SD = 18.9 mm), women had smaller Dmax at rupture (73.4 ± 18.4 mm vs 83.1 ± 18.5 mm, p = 0.003), and smaller neck and iliac diameters compared to men. Aortic size index (ASI) was similar between men and women (4.1 ± 3.1 cm/m^2^ vs 3.8 ± 1.0 cm/m^2^). Fourteen percent of all patients ruptured at Dmax ≤ 60 mm, and a higher proportion of women compared to men ruptured at Dmax ≤ 60 mm: 27% (12/45) vs. 10% (15/147), p = 0.005. Also, a higher proportion of patients with a chronic obstructive pulmonary disease ruptured at Dmax ≤ 60 mm (34.6% vs 14.6%, p = 0.026). Supra-renal aortic size index (14.0, IQR 13.3–15.3 vs 12.8, IQR = 11.4–14.0) and peak wall rupture index (PWRI, 0.35 ± 0.08 vs 0.43 ± 0.11, p = 0.016) were higher for small rAAAs compared to matched iAAAs. Aortic size index, peak wall stress and aneurysm volume did not differ.

**Conclusion:**

More than one tenth of ruptures occur at smaller diameters, women continuously suffer an even higher risk of presenting with smaller diameters, and this must be considered in surveillance programs. The increased supra-renal aortic size index and PWRI are potential markers for rupture risk, and patients under surveillance with these markers may benefit from increased attention, and potentially from timely repair.

## Introduction

The benefit of screening for *abdominal aortic aneurysms* (AAAs) in 65-year old men has been evaluated in several countries and programs, and remains beneficial at prevalence rates of 0.35–0.5%. [1,2]. Despite the introduction of screening programs in the UK and Sweden, up to a third of patients, in particular women and younger men, suffer from rupture under surveillance [3–7]. Women and smokers are at a particularly high risk of rupture [8], but population-based screening in women or younger patients has been deemed not cost-effective [9].

Surveillance in diagnosed patients is based on repeated diameter measurements, which are used as surrogate markers of growth and rupture risk. Several studies have reported that the mean diameter for *ruptured AAAs* (rAAAs) is close to 80 mm, while some aneurysms still rupture prior to reaching the surgical threshold of 50–55 mm [10–12]. The 55 mm threshold in men comes from RCTs based on ultrasound using the inner-to-inner wall diameter measurements, which is rather equivalent to 57–59 mm on computed tomography (CT). This paradox of stated and used diameters is rarely reflected upon in standard care [13,14]. Even if aneurysm diameter growth is reported to be more accurately predicted by aneurysm volume than diameter [15], and biomechanical analysis of AAAs has been shown to outperform diameter measurements in both growth and rupture risk prediction [16,17], diameter is still the gold standard for surveillance and threshold for elective repair [13].

The current state-of-care could potentially be improved by individualizing surveillance protocols to include patient characteristics, aneurysm morphology and biomechanics. Such regimens would enhance the possibility to alter surveillance intervals, and schedule timelier intervention (sooner or later). Several publications identify female AAA patients as holding a higher rupture risk. Fragile morphological features and lower wall strength could be contributing factors [18–21]. An important factor to consider is the aneurysm diameter in association to the *body surface area* (BSA) combined with the infrarenal *Aortic Size Index* (ASI), which is rarely investigated in most materials on rAAAs, although it is likely to influence the overall rupture risk, and could contribute to the understanding of sex-differences [22,23]. The suprarenal diameter could contribute with a morphological assessment of generalized ectatic disease. In general, further efforts should be made to identify patients at high risk during surveillance, especially in groups with an epidemiological overrepresentation of rupture, such as women [24].

The aim of this study was to characterize the morphology of rAAAs in a population-based cohort, with specific consideration to sex-differences. Further, possible morphological and biomechanical determinants of rupture in small AAAs was investigated by comparing patients with ruptured AAAs to small untreated asymptomatic AAAs under surveillance [25].

## Methods

### Stockholm aneurysm rupture cohort

All patients that presented to one of seven emergency departments in Stockholm County and Gotland County with rAAA (as classified by ICD I71.3) between the years 2009–2013 were considered for inclusion. In 2009, this region was inhabited by 2.1 million people, of which 670 000 were 50 years or older [26]. Two-hundred and eighty-three patients were diagnosed with rAAA in the Stockholm rupture cohort. Patients with previous intervention for AAA were excluded. Inclusion criterion for radiological analysis, which 192 patients fulfilled, was an available CT performed at the time of rupture. The basic characteristics of the cohort are previously reported [27].

### Ethical approval and reporting

This study was approved by the Regional Ethics Review Board in Stockholm and complies with the Declaration of Helsinki. Informed consent was not required with reference to the registry-based design. All data were collected from electronic health care records and anonymized at collection. The reporting of this study conforms to the Strengthening the Reporting of Observational Studies in Epidemiology (STROBE) statement.

### Intact aneurysm cohort

For the comparative analysis of ruptured versus intact AAAs, we included a cohort of intact patients (n = 153). These patients were all identified through the outpatient clinic at the Department of Vascular Surgery where they had undergone thin slice CT Angiography (CTA, <3mm) between the years 2009–2013. The mean age of these patients was 72.7±7.4 and 26 (17%) were women. They were then matched according to nearest-neighbor for Dmax, sex and age, with an automatic matching function implemented in the CRAN MatchIt-package [28].

### Radiological analysis

**Fig 1:**
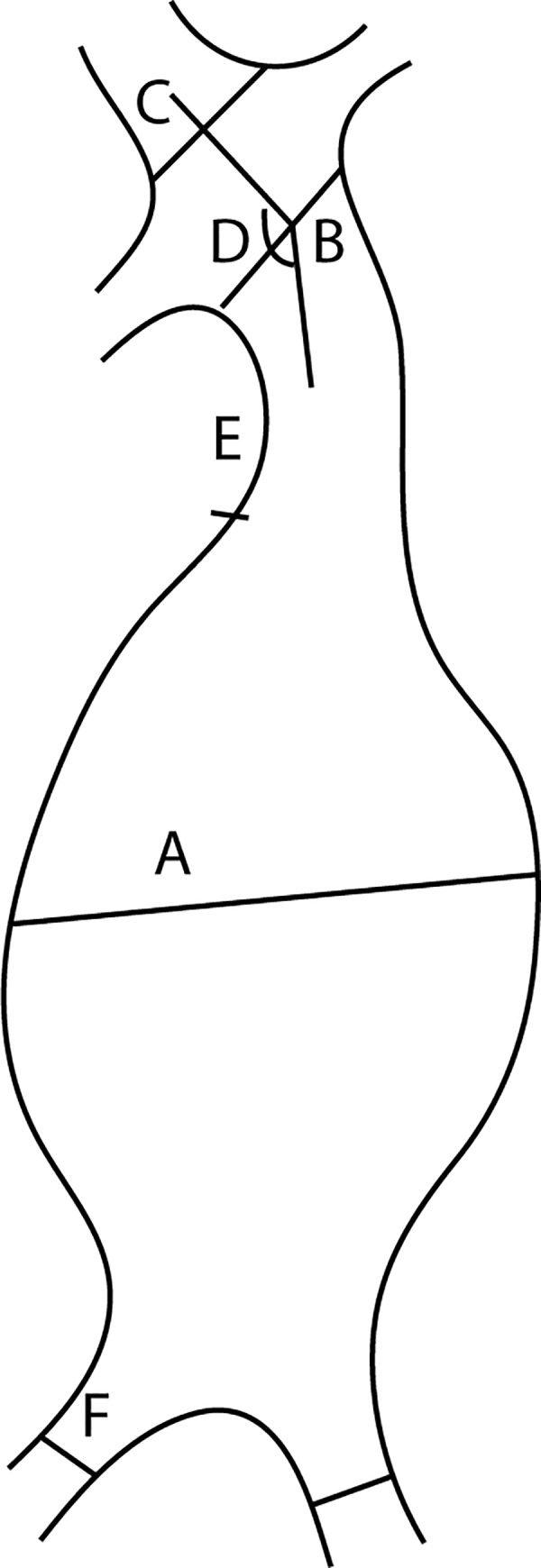
Schematic drawing indicating different centerline-based measurements in the abdominal aortic aneurysms. **A** Maximal diameter, **B** Upper limit of neck, **C** Supra-renal neck, **D** alpha angle, **E** Lower limit of neck, **F** Common iliac diameter.

All CT examinations were exported as DICOM-files from the hospitals picture archiving and communication system and then imported into 3mensio Vascular 8.1 (Pie Medical Imaging B.V, Maastricht, The Netherlands). The neck length, neck diameter, alpha-angle, maximal external diameter (Dmax), maximal left and right common iliac artery diameters were measured according to the St George's Vascular Institute Protocol (Fig 1) [29]. All patients were measured by one of two investigators (A.S or M.L.L). Inter-observer variability of the radiological measurements was acceptable for neck length, neck diameter, Dmax, left common iliac maximal diameter and right common iliac maximal diameter. Mean absolute difference was 3.2 mm, 2.0 mm, 3.3 mm, 1.0 mm and 0.7mm respectively and bias was 1.4±4.5 mm, 0.09±2.4 mm, 0.48±4.54 mm, 0.44±1.13 mm and 0.3±0.9 mm respectively.

A centerline was constructed through the center of each aneurysm. The supra-renal diameter was measured 1 mm proximal to the most proximal renal artery. The neck diameter was measured distal to the most distal renal artery (upper limit of the neck). The neck length was defined as the length along centerline between the upper limit of the neck and the point of maximal aneurysmal dilatation. The alpha angle was measured as the deviation of the aorta 20 mm above and below the upper limit of the neck. Dmax was defined as the diameter at the maximal vessel widening, which was assessed as the largest in stretched vessel view. All diameters were measured perpendicular to the centerline as the mean of the anterio-posterior (AP) and lateral measurements. *Aortic Size Index* (ASI) was calculated as the ratio between Dmax and BSA, estimated according to Du Bois [30], and supra-renal ASI was defined as the ratio of the supra-renal diameter to BSA. An aneurysm was defined as saccular if the dilatation did not involve the entire circumference of the aneurysm, and otherwise as fusiform.

### Biomechanical analysis

Finite element analysis (FEA) was performed using A4 Clinics 5.0 (VASCOPS GmbH, Graz, Austria). The process is detailed elsewhere [31]. In short, a 3D model of the AAA including the intraluminal thrombus (ILT), lumen and vessel wall is semi-automatically reconstructed from a CTA examination. The model is then processed into a hexahedral mesh. Aneurysm tissue properties are modelled as hyperelastic, incompressible and isotropic. Simulations were performed with neutral patient-characteristics. The output is peak wall stress (PWS) which is the highest stress at any point in the aneurysm, and peak wall rupture index (PWRI) which is the highest ratio of wall stress to wall strength.

A group of AAAs with the Dmax smaller than or equal to 60 mm were selected for detailed morphological analysis (“small” rAAA, n = 27). Seven patients were excluded due to too thick slices. Three-dimensional models and volume measurements could be performed in 20/27 patients. Among these, 5 patients were excluded due to lack of intravenous contrast and FEA was performed in 15 of 27 patients.

### Statistical analysis

Continuous data are presented as mean and standard deviation (SD) or median (IQR) for parametric and non-parametric data, respectively. Student’s t-test or Mann-Whitney U test was used to compare continuous variables. Fisher’s exact test and Chi-squared test were used to test differences for categorical variables. For survival analysis, patients were censored at the time of data collection (2016-12-31). Kaplan-Meier curves were used to illustrate survival analysis and univariate Cox proportional hazard regression test was used for comparing groups. Hardman score was calculated [32]. In case of missing data, only complete-cases were analyzed.

P<0.05 was considered as statistically significant. All analyses were performed with the R programming language (R Foundation for Statistical Computing, Vienna, Austria) [33].

## Results

### Patient characteristics and outcome

One-hundred-ninety-two (67.8%) of 283 patients with verified AAA rupture had CT scans that could be retrieved. A larger proportion of patients for whom no CT was available were untreated compared to patients for whom a CT was available (35.2% vs 20.3%, p = 0.007). They were also more likely to have diabetes, but did not differ with respect to age, sex or other patient characteristics (Table 1). Among the untreated patients, 77.4% had decreased levels of consciousness before arrival or were unconscious at arrival to the hospital compared to 50.9% among the treated patients (p < 0.001). Median time until death for untreated patients (n = 72) was one day, and within two days, 86% had died. All untreated patients admitted with rAAA had died within nine days. Survival for treated patients was not different for men and women, but a higher Hardman score, and OSR correlated with worse survival (S1 Supporting Information).

**Table 1:** Basic characteristics for patients with ruptured abdominal aortic aneurysm where CT imaging was available vs no CT imaging available.

	Overall	CT available	CT not available	p-value
n	283	192	91	
Age at rupture—yrs	78.98 (8.96)	79.07 (9.01)	78.79 (8.92)	0.805
Female (%)	69 (24.4)	45 (23.4)	24 (26.4)	0.697
EVAR (%)	59 (28.8)	44 (29.9)	15 (25.9)	0.683
Smoking (%)				0.915
Never	38 (26.4)	28 (25.7)	10 (28.6)	
Current	57 (39.6)	43 (39.4)	14 (40.0)	
Previous	49 (34.0)	38 (34.9)	11 (31.4)	
Diabetes (%)	35 (12.8)	31 (16.8)	4 (4.4)	0.003
COPD (%)	42 (15.3)	32 (17.4)	10 (11.1)	0.239
Heart Disease (%)	102 (37.4)	71 (38.8)	31 (34.4)	0.571
Hypertension (%)	175 (63.9)	120 (65.2)	55 (61.1)	0.596
Hardman Score (%)				0.370
0	42 (14.8)	25 (13.0)	17 (18.7)	
1	130 (45.9)	86 (44.8)	44 (48.4)	
2	84 (29.7)	60 (31.2)	24 (26.4)	
≥3	27 (9.5)	21 (10.9)	6 (6.6)	
Previously known AAA	85 (30.0)	57 (29.7)	28 (30.8)	0.963

EVAR, endovascular aortic repair; COPD, chronic obstructive pulmonary disease. Data are presented as mean (sd) or n (%), percentages are calculated relative to the total number of non-missing data. *CT available group* 7/192 patients have missing data for 1 Hardman score variable and 3/192 have missing for 2 variables. In the *CT not available group* 2/92 have missing data for 1 Hardman score variable and 6/92 have missing data for 2 variables.

### Radiological findings

**Fig 2:**
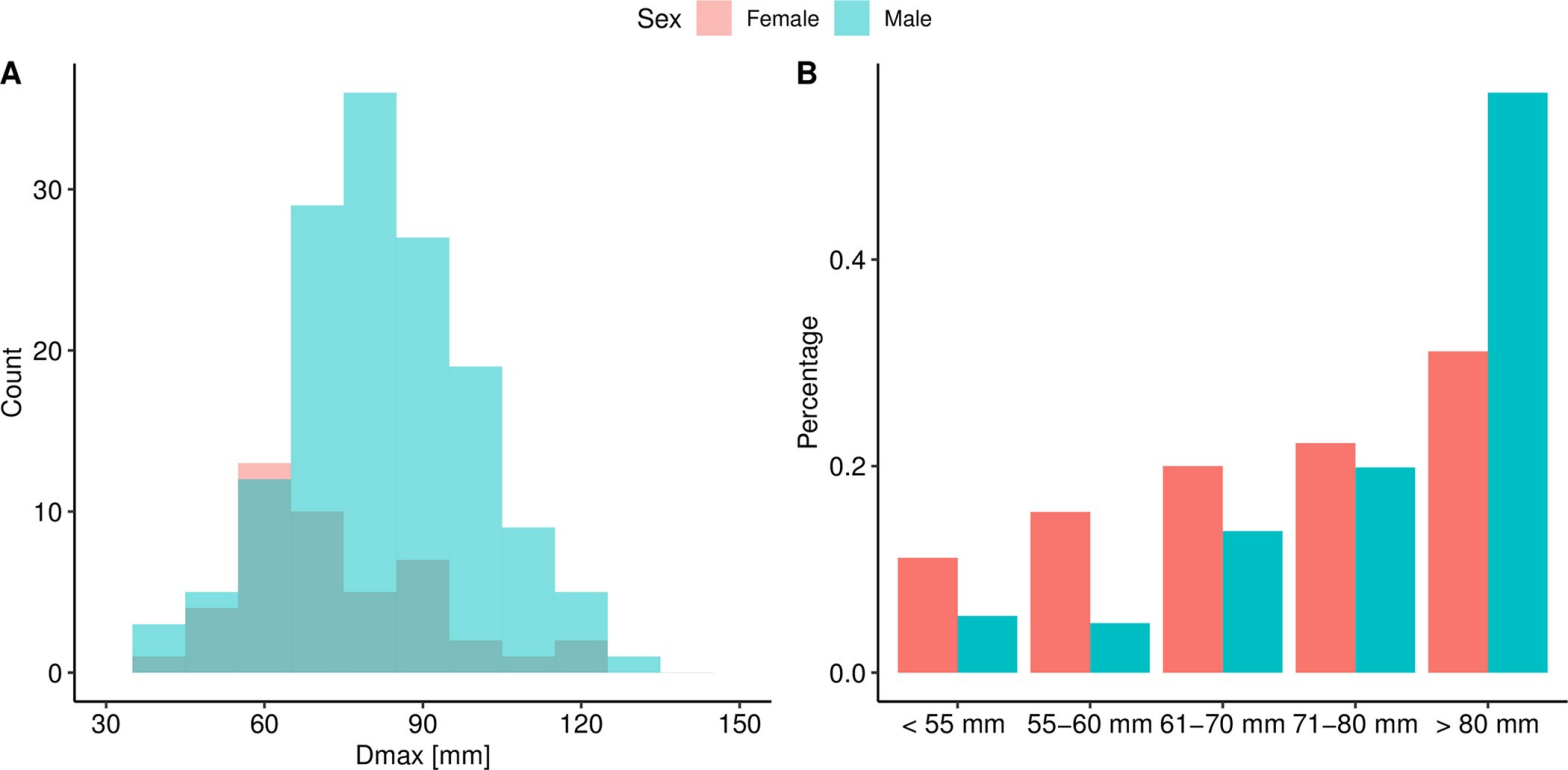
(A) Histograms displaying Dmax at rupture for men and women. (B) Bars indicate the proportions among men and women with ruptured abdominal aortic aneurysms according to Dmax.

**Fig 3:**
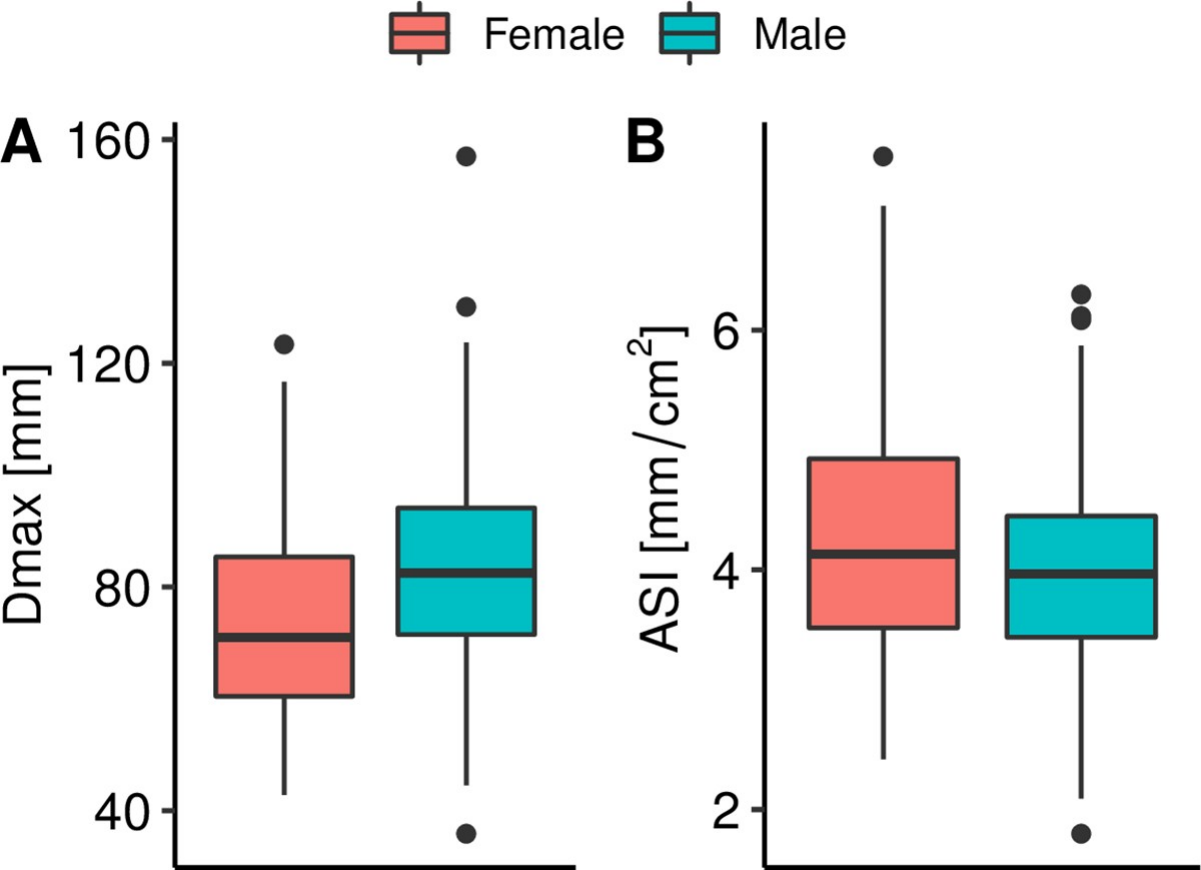
(A) Maximal aneurysm diameter (Dmax) of rAAAs for men and women. (B) Aortic size index (ASI) of rAAAs for men and women. ASI was available for 130 of 192 patients with CT imaging at rupture.

The mean maximum aortic diameter (Dmax) at rupture for all rAAAs with a CT scan (n = 192) was 80.8 mm (SD = 18.9 mm, range 35.9–157.0 mm) (Fig 2A). Twenty-seven patients (14.0%) had a Dmax ≤ 60 mm and 14 (7.2%) had a Dmax ≤ 55 mm. A larger proportion of women compared to men had a Dmax ≤ 60 mm, 27% (12/45) vs 10% (15/147), p = 0.005, and at Dmax ≤ 55 mm (13% vs 5%, p = 0.08) (Fig 2B). The mean Dmax at rupture was smaller for women than men (73.4 ± 18.4 mm vs. 83.1 ± 18.5 mm, p = 0.003) (Fig 3A), but ASI was similar (4.1 ± 3.1 cm/m^2^ vs. 3.8 ± 1.0 cm/m^2^, p = 0.239) (Fig 3B). The neck diameter, the left common iliac diameter and the right common iliac diameter were also smaller for women compared to men, but there were no differences in neck length or alpha angle (Table 2).

**Table 2:** Geometric characteristics of ruptured abdominal aortic aneurysms stratified by sex.

	Overall	Male	Female	p-value
n	192	147	45	
Dmax—mm	80.82 (18.84)	83.08 (18.46)	73.43 (18.37)	**0.002 **
Neck Length—mm	14.50 [1.00, 27.25]	15.00 [1.00, 25.00]	14.00 [6.00, 29.10]	0.638
Neck Diam—mm	25.90 [22.50, 30.81]	27.00 [23.77, 31.52]	22.60 [20.40, 25.80]	**<0.001 **
Alpha Angel—degrees	19.35 [10.90, 33.85]	18.60 [10.05, 33.80]	22.10 [12.20, 35.60]	0.348
Dmax left common iliac—mm	17.70 [15.20, 20.83]	18.05 [15.65, 21.20]	15.30 [12.80, 19.22]	**0.001 **
Dmax right common iliac -mm	17.10 [14.95, 21.85]	17.90 [15.15, 22.55]	16.23 [14.35, 19.45]	**0.040 **

EVAR, endovascular aortic repair; OSR, open surgical repair; Dmax, maximal diameter. Data are presented as mean (sd) or median [IQR].

There was no difference in Dmax at rupture between AAAs that were previously known compared to previously unknown, (median 81.7 mm; IQR 69.9–92.7 mm vs. 78.63 mm; IQR 63.0–89.1 mm, p = 0.15). In total, 5 rAAAs had a saccular morphology (2.6%), this did not differ between rAAAs with Dmax ≤ 60 mm (7%, 2/27) compared to larger AAAs (2%, 3/166), p = 0.144. A higher proportion of patients with a chronic obstructive pulmonary disease (COPD) ruptured at Dmax ≤ 60 mm (34.6% vs 14.6%, p = 0.026). In a multivariate logistic regression model both sex (p<0.001) and COPD (p = 0.037) were independently associated with rupture at Dmax ≤ 60 mm. (S1 Table). Heart disease, hypertension, smoking status and if the aneurysm was known did not differ between small and large rAAAs (Table 3).

**Table 3:** Patient characteristics for small and large ruptured abdominal aortic aneurysms.

	<60 mm	>60 mm	p-value
n	27	165	
Female (%)	12 (44.4)	33 (20.0)	0.011
COPD(%)	9 (34.6)	23 (14.6)	0.026
Diabetes (%)	5 (19.2)	26 (16.5)	0.778
Heart Disease (%)	9 (34.6)	62 (39.5)	0.799
Hypertension (%)	17 (65.4)	103 (65.2)	>0.999
Smoking (%)			0.128
Never	4 (20.0)	24 (27.0)	
Current	5 (25.0)	38 (42.7)	
Previous	11 (55.0)	27 (30.3)	

COPD, chronic obstructive pulmonary disease. Values are expressed as n (%)

### Morphology and biomechanics of rupture in small aneurysms

**Fig 4:**
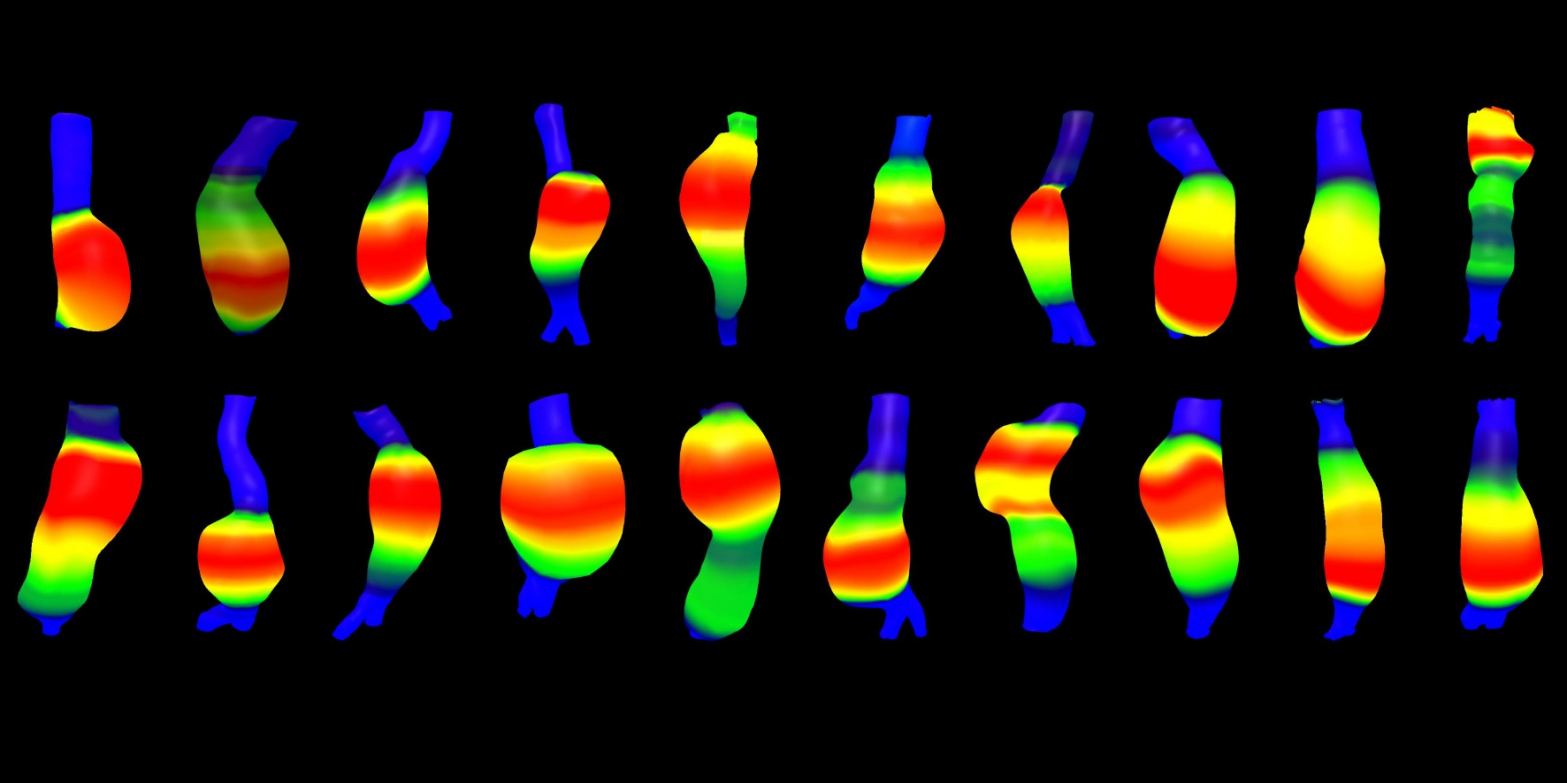
3D-morphology of 20 small (Dmax ≤ 60mm) ruptured AAAs in the Stockholm Population based cohort, colors indicate relative diameter.

The 27 AAAs with Dmax ≤ 60 mm are further on referred to as small rAAAs. Two of these 27 AAAs had a saccular morphology. In seven of the aneurysms it was not possible to construct 3D models. Fig 4 shows the 3D-morphology of 20 small ruptured aneurysms.

The aortic morphological and biomechanical features specific for small rAAAs were compared to Dmax-, age- and sex-matched small iAAAs. There were no differences with regards to Dmax (53.1 ± 5.5 mm vs. 54.5 ± 5.2 mm, p = 0.319), age, and sex (Table 4). BSA did not differ (1.9 ± 0.2 vs 1.8 ± 0.3, p = 0.170). There was a trend towards shorter necks (11.5, IQR = 6.8–32.0 vs 31.6, IQR = 14.0–44.4, p = 0.053) and higher ASI (31.4 ± 6.1 vs 28.4 ± 4.1, p = 0.067) among ruptures. Supra-renal ASI was significantly higher in rAAAs (14.0, IQR 13.3–15.3 vs 12.8, IQR = 11.4–14.0), p = 0.025). Neither total AAA volume, nor PWS differed between the two groups. PWRI, however, was higher for small rAAAs (0.35 ± 0.08 vs. 0.43 ± 0.11, p = 0.016). In a logistic multivariate analysis, both PWRI (p = 0.007) and supra-renal ASI (p = 0.019) were independently significantly associated with the outcome rAAA or iAAA (S2 Table). Other morphological variables did not differ between the iAAAs and the rAAAs (S3 Table).

**Table 4:** Diameter-, sex- and age-matched analysis of small ruptured abdominal aortic aneurysms and asymptomatic controls.

	Asymptomatic AAA	Ruptured AAA	p
n	40	20	
Dmax—mm	53.08 (5.46)	54.54 (5.22)	0.319
Age—years	77.72 (6.67)	78.60 (7.03)	0.647
Female	16 (40.0)	9 (45.0)	0.926
BSA—m^2^	1.89 (0.19)	1.79 (0.27)	0.170
Neck length—mm	31.55 [14.00, 44.35]	11.50 [6.75, 32.00]	0.053
Suprarenal diameter—mm	24.45 [22.03, 26.10]	24.85 [23.27, 27.00]	0.304
ASI mm/m2	28.34 (4.12)	31.39 (6.15)	0.067
Surarenal ASI -mm/m^2^	12.79 [11.44, 13.98]	13.96 [13.33, 15.29]	**0.025 **
Aneurysm volume—cm^3^	156.03 (49.07)	144.39 (32.37)	0.278
PWS—kPa	197.00 (40.26)	216.25 (45.28)	0.162
PWRI—ratio	0.35 (0.08)	0.43 (0.11)	**0.016 **

Dmax, Maximal diameter; BSA, body surface area; ASI, aortic size index; PWS, peak wall stress; PWRI, peak wall rupture index. Values are expressed as mean (sd), median [iqr] or n (%).

## Discussion

This, population-based study, with a detailed morphological analysis, reveal that a considerably higher proportion of women present with smaller AAA at rupture than men. ASI, however, was similar between men and women at rupture. In addition, more patients with COPD were found in the group of patients with smaller diameters. The detailed morphological and biomechanical analysis revealed that saccular morphology is rarely found, even in the cohort of small rAAA and in women. Interestingly, biomechanical and morphological classification (PWRI and supra-renal ASI) could differentiate CT findings from the ruptured versus the asymptomatic aneurysms.

In this study, we considered <60 mm as a small rAAA. In the UK small aneurysm trial (UKSAT), AAAs with Dmax <55 mm were randomized to treatment or surveillance [14]. In the UKSAT, however, anterio-posterior ultrasound was used to measure diameter (inner-to-inner). Relative to perpendicular centerline diameter in CTs, ultrasound may underestimate the diameter by as much as 7.3±7.0 mm [34]. Thus aneurysms, in our study measured by CT as <60 mm would likely represent AAAs that were <55 mm in UKSAT. More women than men suffer from rupture of small aneurysms in our cohort, which confirms what has been previously reported [8]. This may be explained by an aneurysm geometry that increases biomechanical stress [18,19], and a weaker aneurysm wall [20]. Women also had smaller aneurysm necks and smaller common iliac diameters. Our findings, however, indicate that ASI is similar in men and women with ruptured AAAs. These results are consistent with those reported by Lo et al previously, where they reported that women and men have an ASI of 4.1 ± 3.1 cm/m^2^ and 3.8 ± 1.0 cm/m^2^ respectively, compared to our results that show 4.4 ± 1.2 cm/m^2^ and 4.0 ± 0.9 cm/m^2^ [22].

Patients with COPD were also more commonly found in the group of patients with small aneurysms. In a recent meta-analysis, that examined factors for rupture in small AAAs, this was not examined, but there is biological similarity between AAA disease and COPD with matrix destruction [35], and previously decreased forced expiratory volume at 1 s (FEV1) and the presence of COPD have been associated to rupture [36,37].

### Morphology and biomechanics of small rAAA

The aneurysms in the Dmax, age- and sex-matched analysis were similar, also with regard to total volume and PWS. PWS is raised in rAAAs, but the absolute value of the stress varies and AAAs can have several regions of elevated wall stress [31,38,39]. PWRI can by integrating wall strength, theoretically distinguish the relevant stresses and could thus be a better rupture risk predictor. PWRI is, in contrast to PWS, also increased in pre-rupture AAAs [40], and in this study is also increased in small rAAAs. It should be noted that biomechanical analysis with patient neutral constitutive parameters was employed, meaning that the extrapolated wall properties only rely on the aneurysm geometry. Blood pressure is one of the constitutive parameters for the biomechanical analysis, and is likely greatly deranged at rupture, why we chose to omit patient-specific parameters.

Morphologically, there was a non-significant trend towards larger ASI and shorter necks in the ruptured AAAs. Aneurysm volume was similar. In men and women with similar diameters, the supra-renal diameter and the relative dilation between the supra-renal diameter and Dmax is higher for women [23]. Here, despite controlling for Dmax, sex and age, the supra-renal diameter adjusted for BSA (the supra-renal ASI) was higher among ruptures. There may be several explanations for this, but one is that supra-renal ASI may be a marker of more generalized aortic disease.

It has been suggested that small rAAAs are more likely to be saccular [11]. In the 20 patients with 3D-segmented aneurysms, none revealed a saccular morphology, and in total, only a minority of the small rAAAs were saccular. There is conflicting evidence in the literature regarding the rupture risk of saccular aneurysms [11,41]. It should be noted that our results may rather reflect clinical practice paradigms where saccular aneurysms are recommended to be treated at smaller diameters [13]. It is clear that small aneurysms can rupture, regardless of saccular or fusiform morphology, and that saccular morphology does not appear to be the major contributing factor in rupture of either small or large AAAs.

Surveillance is reported to be safe in men with screening-detected AAAs. A recent study showed that the annual rupture risk in surveillance was below 0.5% for these patients [42]. Further, recent evidence indicates that rupture rates in large aneurysms are lower than what has previously been reported [43]. Here we observe that the median diameter for male patients at rupture was 83.1 mm. This should put into question the current treatment policy, where for some male patients the intervention threshold appears unnecessarily low. Women, however, rupture more frequently at smaller diameters, and for women with a low surgical risk, surveillance must be rigorous. Here we report, as has previously been reported by Lo et al, that ASI is similar in men and women at rupture [22]. If an aneurysm definition that is based on ASI, instead of Dmax, is used, the prevalence of AAAs in women and men are almost equal [44]. The optimal policy for personalized surveillance cannot be suggested based on the current study, but factors such as ASI and PWRI should further be investigated, preferably in a prospective trial. Contemporary analysis on population-based screening in men confirms the cost effectiveness, and reduction in AAA-related and all-cause mortality, which is not shown in women [45]. It is however cost effective to invite targeted risk groups, such as first-degree relatives, which would also include women at high risk [46]

This study suffers from potential selection bias, since a fraction of patients with rAAAs do not reach the hospital, are left untreated, or do not undergo CT. Some CT scans could also not be retrieved. We, however, found no major differences in patient characteristics between patients, aside from a larger proportion being untreated, which likely explains the decision to refrain from imaging. The study cohort does however represent a typical population of patients admitted to hospital with rAAA, both untreated and treated and would therefore be a reasonably generalizable cohort of every-day practice in most vascular services in the western world. A further limitation is the potential change in geometry and biomechanical parameters at rupture. It has not been proven if biomechanical findings are different or similar between pre-rupture aneurysms and ruptured aneurysms. In the quest for an improved prediction of rupture risk, the pre-rupture aneurysms are of course the clinically relevant entity. As a consequence of this and the small sample size among the ruptured and non-ruptured AAAs, these results must be seen as indicative rather than conclusive.

## Conclusions

In this population-based analysis of rAAAs, a high proportion of patients had small aneurysms at rupture, more women and patients with COPD were represented in these cases. Saccular morphology was not an influential factor among small or large aneurysms. The previously reported high proportion of patients admitted with rAAA, already known by the healthcare system, stresses the importance to identify predictors that could improve surveillance strategies.

Our results support, as has been shown by others, that ASI could be such a valuable tool in the individualized evaluation when considering timing of surgery, especially in women.

The higher PWRI and supra-renal ASI in the small rAAA indicates that these variables also should be further evaluated as possible predictors.

## Supporting information

S1 Supporting Information Survival after treatment of ruptured AAAs in the stockholm aneurysm rupture cohort. (DOCX)

S1 Table Multivariate logistic regression of COPD and sex as predictors of dmax ≤ 60 mm at rupture. (DOCX)

S2 Table Multivariate logistic regression of supra-renal ASI and PWRI as predictors of rupture in small rAAAs compared to iAAA. (DOCX)

S3 Table Morphological variables in 40 asymptomatic and 20 ruptured AAAs, matched by Dmax, sex and age. (DOCX)
