# Supplementary material for: A large proportion of patients with small ruptured abdominal aortic aneurysms are women and have chronic obstructive pulmonary disease
Source: PLoS One. 2019 May 28;14(5):e0216558. doi: 10.1371/journal.pone.0216558 (PMC6538142; doi:10.1371/journal.pone.0216558)
Supplement: S1 Supporting Information — (DOCX) [file pone.0216558.s001.docx]

**Survival after abdominal aortic aneurysm rupture**

Among treated patients with CT-imaging at rupture, there was no difference in survival for men compared to women (hazard ratio, HR = 0.87, 95% CI 0.52-1.45, p = 0.59) (Fig 5). Patients that were treated by OSR had a worse prognosis compared to patients treated by EVAR (HR = 0.59, 95 % CI 0.35-0.97, p = 0.039). A higher Hardman score was associated with worse survival (≤1 vs ≥2, HR = 1.23, 95% CI 1.07-1.42, p = 0.005), and there was a similar trend for older patients (HR = 1.53, 95% CI 0.99-2.38, p = 0.055). Patients with smaller aneurysms had a trend towards worse survival (HR = 0.67, 95% CI 0.43-1.03, p = 0.067). There was no difference in survival among treated patients depending on whether CTs could be retrieved or not (HR = 0.75, 95% CI 0.51-1.09, p = 0.141).


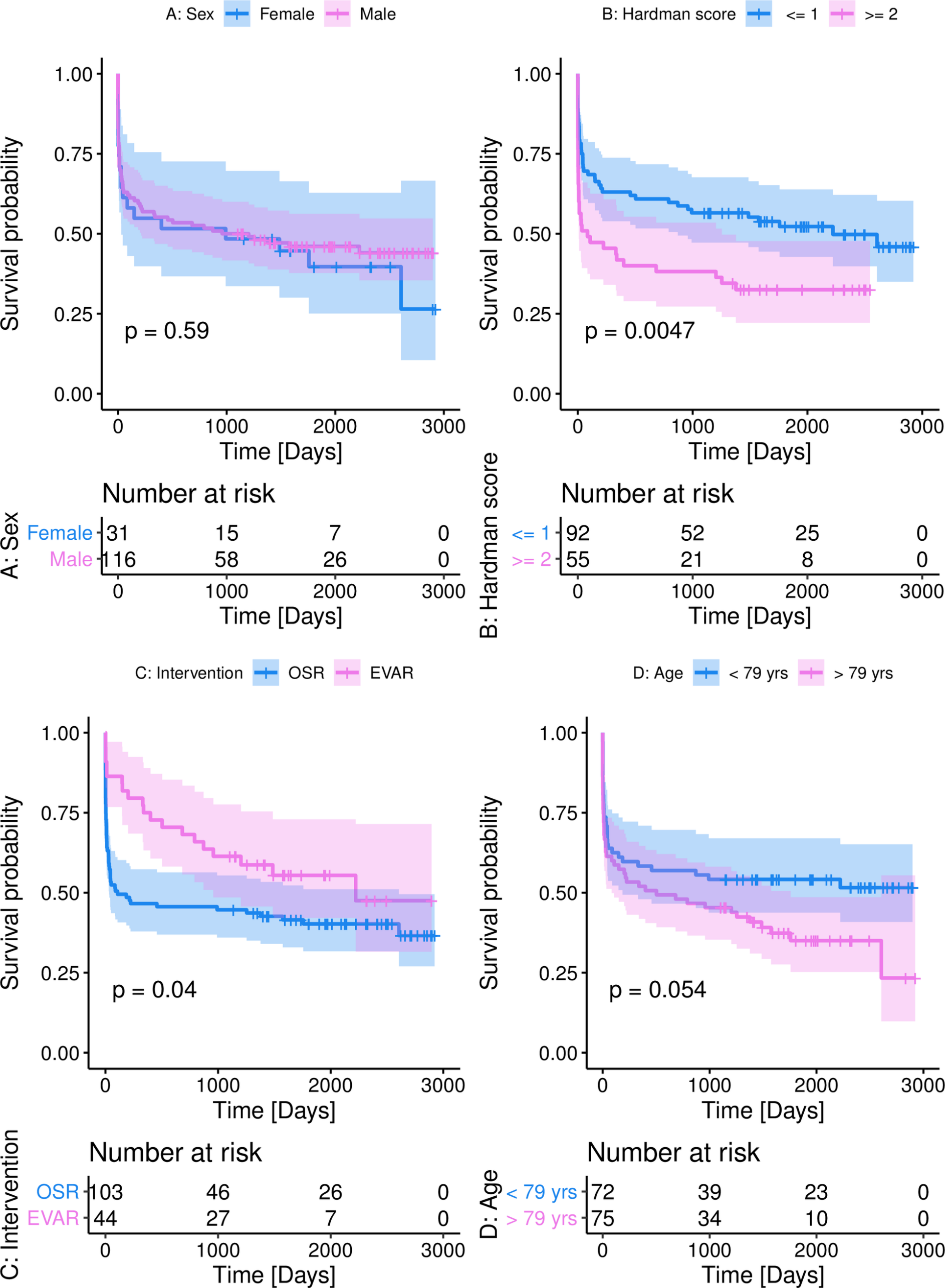


Kaplan-Meier curves for survival after AAA rupture for treated patients with CT-verified rupture, 95% confidence intervals and risk tables. Stratified by (A) Sex, (B) Hardman score, (C) Open surgical repair (OSR) or endovascular aortic repair (EVAR), and (D) Age.
