# Supplementary material for: A large proportion of patients with small ruptured abdominal aortic aneurysms are women and have chronic obstructive pulmonary disease
Source: PLoS One. 2019 May 28;14(5):e0216558. doi: 10.1371/journal.pone.0216558 (PMC6538142; doi:10.1371/journal.pone.0216558)
Supplement: S1 Table — (DOCX) [file pone.0216558.s002.docx]

|  | Estimate | Std. Error | P-value |
| --- | --- | --- | --- |
| Intercept | -2.41 | 0.31 | <0.001 |
| COPD | 1.01 | 0.48 | 0.037 |
| Sex | 1.17 | 0.45 | <0.001 |
