# Supplementary material for: A large proportion of patients with small ruptured abdominal aortic aneurysms are women and have chronic obstructive pulmonary disease
Source: PLoS One. 2019 May 28;14(5):e0216558. doi: 10.1371/journal.pone.0216558 (PMC6538142; doi:10.1371/journal.pone.0216558)
Supplement: S2 Table — (DOCX) [file pone.0216558.s003.docx]

|  | Estimate | Std. Error | P-value |
| --- | --- | --- | --- |
| Intercept | -12.51 | 3.68 | <0.001 |
| PWRI | 12.54 | 4.69 | 0.007 |
| Supra-renal ASI | 0.48 | 0.19 | 0.019 |
