# Supplementary material for: A large proportion of patients with small ruptured abdominal aortic aneurysms are women and have chronic obstructive pulmonary disease
Source: PLoS One. 2019 May 28;14(5):e0216558. doi: 10.1371/journal.pone.0216558 (PMC6538142; doi:10.1371/journal.pone.0216558)
Supplement: S3 Table — (DOCX) [file pone.0216558.s004.docx]

|  | **Asymptomatic AAAs (n=40)** | **Ruptured AAAs (n=20)** | **p-value** |
| --- | --- | --- | --- |
| **Neck diameter [mm]** | 24.1 ± 4.7 | 24.5 ± 6.9 | 0.962 |
| **Alpha angel [degrees]** | 13.6 (8.8-25.7) | 18.5 (11.1-24.4) | 0.748 |
| **Right common iliac [mm]** | 16.1 ± 4.0 | 15.8 ± 2.0 | 0.826 |
| **Left common iliac [mm]** | 16.1 ± 4.0 | 15.9 ± 3.3 | 0.676 |
